# Molecular characterization and analysis of high-level multidrug-resistance of *Shigella flexneri* serotype 4s strains from China

**DOI:** 10.1038/srep29124

**Published:** 2016-07-04

**Authors:** Chaojie Yang, Peng Li, Xiujuan Zhang, Qiuxia Ma, Xianyan Cui, Hao Li, Hongbo Liu, Jian Wang, Jing Xie, Fuli Wu, Chunyu Sheng, Xinying Du, Lihua Qi, Wenli Su, Leili Jia, Xuebin Xu, Jiayong Zhao, Shengli Xia, Na Zhou, Hui Ma, Shaofu Qiu, Hongbin Song

**Affiliations:** 1 Institute of Disease Control and Prevention, Academy of Military Medical Sciences, Beijing 100071, China; 2 Beijing Chaoyang Hospital, Capital Medical University, Beijing 100043, China; 3 Shanghai Municipal Centre for Disease Control and Prevention, Shanghai 200336, China; 4 Henan Provincial Centre for Disease Control and Prevention, Zhengzhou 450016, China; 5 Health Bureau, Logistics Support Department of Central Military Commission, Chinese PLA, Beijing 100038, China

## Abstract

To conduct the first comprehensive analysis of *Shigella flexneri* serotype 4s, a novel serotype found in 2010, we identified 24 serotype 4s isolates from 1973 shigellosis cases in China (2002–2014). The isolates were characterized by single nucleotide polymorphism (SNP) phylogenetic analysis, pulsed-field gel electrophoresis (PFGE) and multilocus sequence typing (MLST) to determine their genetic relatedness, and analysed further for their antimicrobial susceptibilities and antimicrobial resistance determinants. The PFGE and SNP phylogenetic analyses suggest that *S. flexneri* serotype 4s strains are derived from multiple serotypes, including two predominant serotypes in China: serotype X variant and serotype II. Three new sequence types were identified by MLST. All isolates were resistant to ticarcillin, ampicillin and tetracycline, with high-level resistance to third-generation cephalosporins. Notably, all the isolates were multidrug resistant (MDR), with the highest levels of resistance observed for eight antimicrobials classes. Most isolates contain various antimicrobial resistance determinants. In conclusion, we found that serotype 4s isolates have multiple evolutionary sources, diverse biochemical characteristics and genomes, and highly prevalent multidrug resistance and antimicrobial-resistant determinants. With few clinical treatment options, continuous monitoring and timely intervention against this emerging MDR serotype is essential. The possibility that serotype 4s will become the next predominant serotype exists.

Shigellosis, caused by *Shigella*, is a major global public health concern, with nearly 167 million cases and over a million deaths occurring annually worldwide^1^, mostly in children under 5 years of age. Shigellosis is prevalent in under developed countries because of poor sanitation^2^. Based on the O-antigen structures of the membrane-associated lipopolysaccharide, *Shigella* can be divided into four species or subgroups^3^: *Shigella flexneri, S. dysenteriae, S. boydii* and *S. sonnei*. Among these, *S. flexneri* is the most common cause of endemic shigellosis, and is the most frequently isolated species in mainland China^1^,^4^. Based on its serology, *S. flexneri* can be further subdivided into at least 15 serotypes (1a, 1b, 1c, 2a, 2b, 3a, 3b, 4a, 4b, 5a, 5b, Y, X, Xv and 6)^5^,^6^.

Continued selective pressure by a variety of antibiotics has resulted in bacteria developing resistance mechanisms that lead to multidrug resistance (i.e., resistance to three or more antimicrobial classes)^7^. In recent years, the rapid development of antimicrobial resistance amongst *Shigella* species has been reported worldwide^8^,^9^. Multidrug-resistant (MDR) *Shigella* isolates narrow the choice of effective antimicrobials^10^,^11^, thereby decreasing the reliable treatment options for Shigellosis. This has become a major obstacle for the control of shigellosis^12^.

Molecular characterization of *S. flexneri* can help us to identify its specific subtypes more accurately and determine the real source of a novel serotype and enable investigation of the infectious source during pathogen outbreaks^13^. Through molecular characterization of a pathogen in different geographic regions, we can determine its range of dissemination, the emergence of variants, and its closely related strains in one lineage^14^. Analysis of antibiotic resistance and resistance genes can help us to understand drug resistance mechanisms, thereby helping to guide clinical treatment of the disease.

Because of antigenic variation, several novel *S. flexneri* serotypes (such as 1c, 1d and 4s) have emerged in recent years^15^,^16^,^17^. *Shigella flexneri* serotype 4s was first described in 2010^17^, and is a sub-serotype of serotype 4, displaying atypical agglutination patterns. When tested with antisera from Denka Seiken Co. Ltd., Japan, serotype 4s reacts with monovalent IV-type antisera but not with any group-specific antisera. When tested with antisera from Reagensia AB (Sweden), it exhibits agglutination with the serogroup B-specific antibody MASF B and the group antigen-specific antibody MASF IV-1, but not with MASF IV-2. Notably, the first *S. flexneri* serotype 4s strain described was MDR^17^, but this may be not represent a true picture of serotype 4s strains because only one strain was identified and tested at that time and no further molecular characterization or antimicrobial resistance profiling of this serotype has been conducted since. Therefore, the objectives of this study were to identify all the *S. flexneri* serotype 4s isolates available to us during our routine surveillance of shigellosis in the eastern, western, southern, northern and central regions of China, to examine the antimicrobial resistance patterns and antimicrobial resistance determinants of those isolates, and to perform molecular characterization of them to determine the relationships between *S. flexneri* serotype 4s and other subtypes.

## Results

### Bacterial isolation and biochemical characterization

Twenty-four *S. flexneri* serotype 4s isolates were identified amongst the 1973 *S. flexneri* isolates collected from the eastern, western, southern, northern and central regions of China during our 13-year routine surveillance (from 2002 to 2014) of shigellosis. This serotype was relatively dispersed across China according to our surveillance results, suggesting that *S. flexneri* serotype 4s is still relatively rare in China, and that there is not currently an outbreak of this serotype. Biochemical characterization showed that the 4s isolates displayed inconsistent biochemical features (Table 1). Four isolates from Shanghai and three isolates from Guangxi produced indole, one isolate (SH4s01) could not ferment melibiose but could ferment sorbitol, and SH4s04 also could not ferment melibiose. These features differ from those of the first-reported 4s isolate^17^, which could ferment melibiose, but could not produce indole and did not use sorbitol.

### Pulsed-field gel electrophoresis (PFGE) and multilocus sequence typing (MLST) analyses

**Figure 1:**
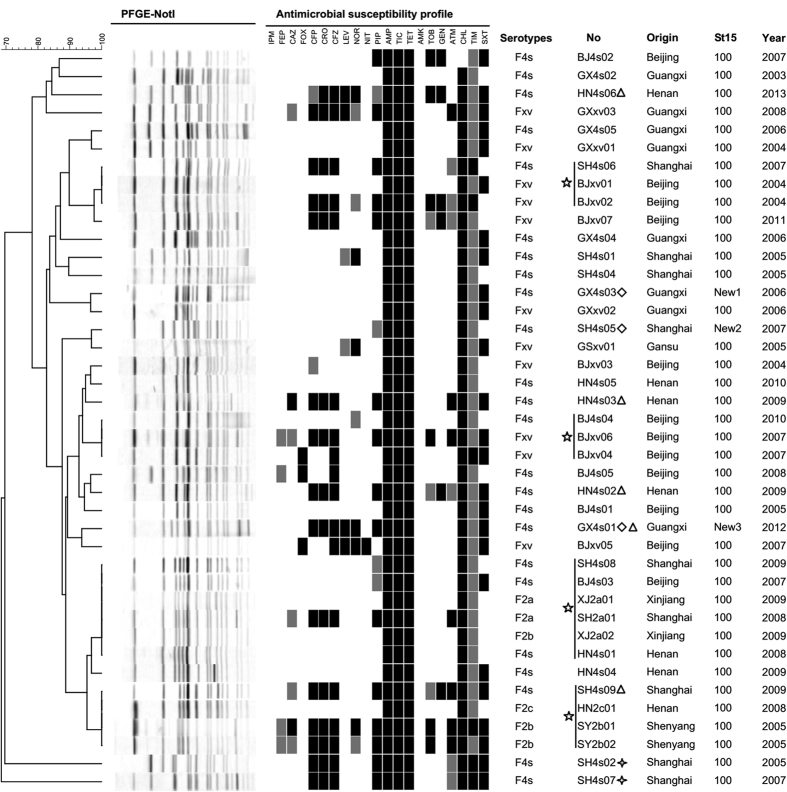
PFGE profiles, antimicrobial susceptibility profiles and sequence types (STs) of *Shigella flexneri* serotype 4s, 2 and xv isolates. The serotype, origin, strain number, year of isolation and ST are listed after each PFGE profile. AMK, amikacin; AMP, ampicillin; ATM, aztreonam; CHL, chloramphenicol; CAZ, ceftazidime; CFP, cefoperazone; CFZ, cefazolin; CRO, ceftriaxone; FEP, cefepime; FOX, cefoxitin; GEN, gentamicin; IPM, imipenem; LVX, levofloxacin; NIT, nitrofurantoin; NOR, norfloxacin; PIP, piperacillin; SXT, trimethoprim/sulfamethoxazole; TET, tetracycline; TIC, ticarcillin; TIM, ticarcillin/clavulanic acid constant 2; TOB, tobramycin. Black indicates “resistant”, grey indicates “intermediate susceptibility” and blank indicates “sensitive”. Stars represent 4s strains whose PFGE patterns are identical. Four cornered stars represent 4s strains whose PFGE patterns are very different from the others. Diamonds represent 4s strains with new MLST types. Triangles represent 4s strains with resistance to at least 7 antibiotic classes.

*S. flexneri* serotype 4s was shown previously to have evolved from the *S. flexneri* serotype X variant (SFxv)^17^. To compare the genetic similarity and verify the evolutionary relationship between these two serotypes, PFGE was performed (Fig. 1). The banding profiles of several of the serotype 4s isolates shared more than 90% similarity with that of SFxv. We also compared the profiles of *S. flexneri* serotype 4s isolates with those of other *S. flexneri* serotypes (1a, 1b, 1c, 2a, 2b, 2c, 4a, 4b, X, Y and 6). Surprisingly, there were two clusters containing *S. flexneri* serotype 4s and *S. flexneri* serotype II (2a, 2b and 2c) isolates each with a similarity score of 100%. This result suggested that *S. flexneri* serotype 4s may have evolved from *S. flexneri* serotype II, indicating multiple evolutionary sources. Several *S. flexneri* serotype 4s isolates (such as SH4s02 and SH4s07) showed distinct banding profiles or low similarities with other strains, suggesting a high level of genetic diversity among the *S. flexneri* serotype 4s strains. There was no significant association between the geographical areas, years of isolation or resistance profiles of isolates that shared similar PFGE patterns.

The MLST results showed that sequence type (ST) 100 was predominant among the *S. flexneri* isolates (Fig. 1). Three STs were found in the 4s isolates (Supplementary Text S1). GX4s03 was designated as a new type of isolate because its rpoS gene, which is most similar to rpoS15, contains a new point mutation. In the ropS gene of GX4s01, a 19-bp sequence was inserted, making this gene contain a new allele sequence. Without the inserted sequence, the GX4s01 ropS sequence is identical to allele rpoS15, indicating that this new allele is also a mutation based on rpoS15. We attributed one new ST, SH4s05, to the new allele combination of 15 housekeeping genes. The SH4s05 allele number for rpoS is 64, while the other allele number is consistent with the ST100 number and this new allele combination is not recorded in the MLST database. All of the new DNA sequences and new allele combinations have been submitted to the MLST database (http://www.shigatox.net/ecmlst/cgi-bin/index).

### Phylogenetic analysis

We sequenced the whole genomes of the 24 *S. flexneri* serotype 4s strains and performed a SNP phylogenetic analysis based on the sequences. To evaluate the similarity between serotype 4s and other serotypes, we selected and downloaded the whole genome sequences of 35 strains, including most of the *S. flexneri* serotypes in the *Shigella* Genome Sequencing Consortium collection^14^ previously analysed and found to be distributed in seven phylogenetic groups (PG1-PG7)^14^.

**Figure 2:**
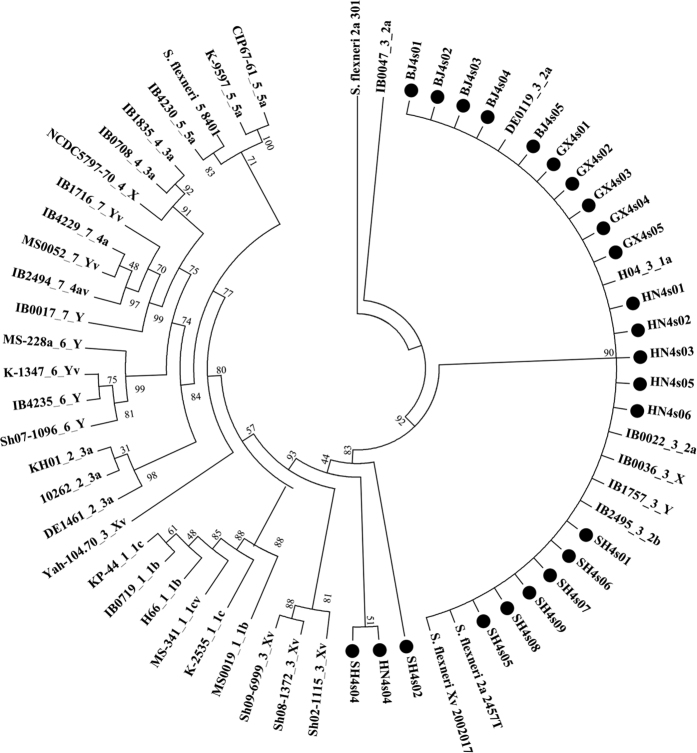
Phylogenetic analysis of *Shigella flexneri* serotype 4s and other *S flexneri* serotypes. The maximum likelihood tree is generated from core SNPs using kSNP^56^ for the 24 *S. flexneri* 4s strains sequenced, 4 standard strains of *S. flexneri* and 35 strains from the Shigella Genome Sequencing Consortium collection^14^. The 24 *S. flexneri* 4s strains are highlighted by black circles. The names of the 35 strains are composited by their origin number, phylogenetic group number and *S. flexneri* serotype.

The phylogenetic tree showed that most of the serotype 4s strains fell within one group mixed with PG3 strains such as the *S. flexneri* serotypes 1a, 2a, 2b, X, Xv, Y and Yv (Fig. 2), suggesting a high level of genome similarity among them^14^. This result is consistent with our PFGE results in that the patterns of serotype 4s show similarity with those of serotype II and Xv. Only three isolates (SH4s02, HN4s04 and SH4s04) fell outside the main clusters, showing independent origins. Although SH4s02 and SH4s07 had distinct PFGE typing patterns with others, the phylogenetic tree revealed that SH4s07 is embedded in the main cluster. This inconsistence indicated that SH4s07 shared similar core regions but different restriction enzyme sites with other serotype 4s strains.

To get more information of the subtle differences among serotype 4s strains, we also performed a SNP phylogenetic analysis only based on the whole genome sequences of the 4s serotype (Supplementary Figure S1). More shared core SNPs were analysed compared with Fig. 2 because of the close relationships among these strains. So although HN4s04 and SH4s04 are close in Fig. 2, they are distinguishable in Supplementary Figure S1. Overall, the phylogenetic tree become more diverse, and the phylogenetic tree showed that these 24 strains were grouped into 3 distinct clusters, suggesting possible multiple origins of 4s serotype.

### Antimicrobial resistance in the *S. flexneri* 4s isolates

**Figure 3:**
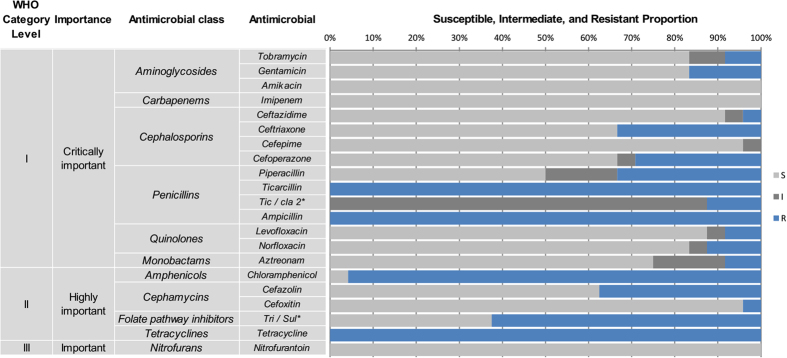
Antimicrobial resistance pattern for *Shigella flexneri* serotype 4s. The classifications “WHO Category Level” and “Importance” are based on the classification determined at the 2011 WHO (World Health Organization) meeting in Oslo, Norway^20^. The antimicrobial agents were categorized as critically important, highly important or important based on two criteria: (1) their use as sole therapy or one of the few alternatives to treat serious human disease, and (2) their use to treat disease caused by organisms that may be transmitted via non-human sources or diseases caused by organisms that may acquire resistance genes from non-human sources. Antimicrobial agents are considered critically important if both criteria (1 and 2) are applicable. Antimicrobial agents are highly important if criterion 1 or 2 is applicable. Antimicrobial agents are important if neither criterion is applicable. TIC/CLA 2*: ticarcillin/clavulanic acid constant 2; TRI/SUL*: trimethoprim/sulfamethoxazole.

The minimum inhibitory concentrations (MICs) and antimicrobial resistance patterns of the 24 *S. flexneri* serotype 4s isolates for 21 antimicrobials are shown in Figs 1 and 3. All of the isolates were resistant to ticarcillin, ampicillin and tetracycline, followed by chloramphenicol (23, 96%), trimethoprim/sulfamethoxazole (15, 63%), cefazolin (9, 38%), ceftriaxone (8, 33%), piperacillin (8, 33%), cefoperazone (7, 29%), gentamicin (4, 17%), ticarcillin/clavulanic acid constant 2 (3, 13%), norfloxacin (3, 13%), tobramycin (2, 8%), levofloxacin (2, 8%), aztreonam (2, 8%), cefoxitin (1, 4%), and ceftazidime (1, 4%). No isolates were resistant to imipenem, cefepime, amikacin or nitrofurantoin.

Among the *S. flexneri* serotype 4s isolates, 33% were resistant to at least one of the third-generation cephalosporins (ceftazidime/ceftriaxone/cefoperazone). The first isolate showing cephalosporin resistance was obtained in 2005, and cephalosporin resistance amongst the *S. flexneri* serotype 4s isolates over the study period was as follows: 2005 (1/4 isolates), 2007 (2/5), 2009 (3/5), 2012 (1/1), 2013 (1/1) (Fig. 1). However, because of the small number of isolates per year, we could not discern a significant linear trend for this serotype in relation to cephalosporin resistance. Two isolates, HN4s06 and GX4s01, were resistant to both third-generation cephalosporins and fluoroquinolones.

Notably, all 24 isolates were MDR. The isolates showed 12 distinct antibiotic-resistance profiles, dominated by resistance to tetracycline/ticarcillin/ampicillin/chloramphenicol (TET/TIC/AMP/CHL; 7/24, 29.0%), followed by tetracycline/ticarcillin/ampicillin/chloramphenicol/trimethoprim and sulfamethoxazole (TET/TIC/AMP/CHL/SXT; 6/24, 25.0%). Two isolates (SH4s09 and HN4s06) were resistant to eight antimicrobial classes, including 11 individual drugs, while three isolates (HN4s02, HN4s03, and GX4s01) were resistant to seven classes (Table 2). These results suggest that multidrug resistance is common amongst *S. flexneri* 4s isolates, unlike the other serotypes we investigated where such a high MDR prevalence was not observed (data not shown).

### Molecular analysis of antimicrobial resistance genes and integrons

All 24 isolates were tested for the presence of AMR (antimicrobial resistance) determinants and integrons, including *bla*_VIM_*, bla*_NDM_*, bla*_SHV_*, bla*_TEM_*, bla*_OXA_*, bla*_CTX-M_*, qnrS*, *aac*(*6*′)*-Ib-cr*, *intI1* and *intI2*. Point mutations within *gyrA*, *gyrB*, *parC* and *parE* were also examined (Table 3). All isolates were negative for *bla*_SHV_*, bla*_VIM_ and *bla*_NDM_; however, all 24 isolates contained both *bla*_TEM-1_ and *bla*_OXA-1_. Seven isolates contained *bla*_CTX-M-14_ or *bla*_CTX-M-79_, and these isolates showed a high degree of resistance to the β-lactamase antibiotics ceftriaxone, cefoperazone and cefazolin. Three isolates carried the following plasmid-mediated quinolone resistance (PMQR) genes: BJ4s03 and GX4s01 carried *qnrS*, and HN4s06 carried both *qnrS* and *aac*(*6*′)*-Ib-cr*. Only two isolates showed resistance to levofloxacin and norfloxacin (quinolone antibiotics) based on the antimicrobial susceptibility testing results, although none of the isolates contained *qnrA*, *qnrB* or *qnrD*. Analysis of point mutations in the quinolone resistance-determining regions (QRDRs) showed that all isolates contained the *gyrA* Ser83Leu and His211Tyr substitutions, along with the *parC* Ser80Ile mutation. Isolate SH4s09 also contained a Ser83Asn substitution in *gyrA* (Table 3). No point mutations were found in *gyrB* or *parE*. All isolates harboured class 1 integrons with *bla*_OXA-1_ + *aadA1*2 gene cassettes, and two isolates from Henan harboured *dfrA17* + *aadA5* or *aacA4* + *cmlA1* gene cassettes. All isolates also contained class 2 integrons with *dfrA1* + *sat1* + *aadA1* gene cassettes.

## Discussion

The emergence of novel and atypical bacterial serotypes in nature is attributed to serotype conversion, which often occurs in response to the protective host immune response^18^. Since the 1990s, several new *S. flexneri* serotypes (e.g., 1c and SFxv) have emerged and become the most prevalent serotypes in some countries^6^,^19^. SFxv first appeared in Henan Province, China, in 2001, and was one of the predominant serotypes in Shanxi, Gansu, and Anhui Provinces from 2002 to 2006^6^,^20^. Data on the prevalence of *S. flexneri* serotypes causing shigellosis in mainland China from 2001 to 2010 suggest that SFxv is the second most predominant serotype after serotype 2a^21^. *S. flexneri* serotype 4s, a novel serotype identified in 2010, is believed to have evolved from serotype SFxv^17^. However, our results based on the SNP phylogenetic and PFGE analyses showed that this novel serotype is not only derived from serotype SFxv but also from *S. flexneri* serotype II (including serotypes 2a, 2b and 2c). A previous study reported that Yv, another new *S. flexneri* serotype, is derived from serotypes Y, SFxv and 2a^22^, suggesting that novel serotypes may be more likely to evolve from predominant serotypes. In addition, the SNP phylogenetic and PFGE analyses showed that *S. flexneri* serotype 4s strains have high genome diversity, suggesting that this serotype may also be derived from other serotypes such as 1a, X, Y or Yv.

The small number of *S. flexneri* serotype 4s isolates among the 1973 *S. flexneri* shigellosis isolates suggested there was no widespread epidemic of this serotype in China. MDR acquisition might explain this finding because drug resistance is often associated with a fitness cost, which is usually related to the replication and maintenance of AMR determinants in bacteria^23^. This fitness cost may result in infections of longer duration in a host and decreased transmission rates in a population. Although this factor may limit a widespread epidemic of *S. flexneri* serotype 4s, the extent to which *S. flexneri* serotype 4s is prevalent in the human population is probably influenced by many factors. Consequently, continuous surveillance of *S. flexneri* serotype 4s is very necessary. First, the present data indicate that serotype 4s evolved from *S. flexneri* serotype II and SFxv strains, which are the predominant serotypes in China. Therefore, *S. flexneri* serotype 4s strains still have the potential to cause an epidemic because of limited host immunity to their new surface antigens. Second, all of the isolates examined to date are MDR, with some isolates displaying resistance to eight antibiotic classes. These isolates have high levels of resistance to the antibiotics classed as “critically important” by the World Health Organization^24^ (i.e., ampicillin, ceftriaxone and ticarcillin). Hence, once an epidemic or outbreak occurs, few effective antimicrobials would be available to clinicians, making infections more difficult to treat.

Analysis of antibiotic resistance genes can help us to understand the fundamental factors leading to bacterial resistance, and assist in developing measures to prevent resistance. In our research, all of the *S. flexneri* serotype 4s isolates contained both *bla*_TEM-1_ and *bla*_OXA-1_. The OXA-1 β-lactamase has a high level of hydrolytic activity against oxacillin and cloxacillin, and confers resistance to ampicillin and cephalothin^25^, while TEM-1 confers resistance to penicillins and the early cephalosporins. *bla*_TEM-1_ is present in almost all extended-spectrum β-lactamase (ESBL)-producing isolates. It is constantly evolving and has generated a number of descendent alleles that confer resistance to most β-lactamase antibiotics^26^. In recent years, these two resistance genes have been found to exist at high frequencies in *Shigella* and other *Enterobacteriaceae*. They are clinically important because they confer resistance to the first generation cephalosporin antibiotics such as ampicillin, which was the principal antibiotic used to treat shigellosis decades ago^27^. Ampicillin resistance in *Shigella* has forced clinicians to seek new effective agents for the treatment of shigellosis. According to the recent recommendations of IDSA (Infectious Diseases Society of America), ceftriaxone (a third generation cephalosporin) and quinolones such as ciprofloxacin and norfloxacin are the treatment options for shigellosis^28^. In addition to *bla*_TEM-1_ and *bla*_SHV_, *bla*_CTX-M_ has been associated with ESBL-producing strains since 1995^29^. It shares less than 40% identity with *bla*_TEM_ and *bla*_SHV_, and comprises a relatively heterogeneous family of 40 members, which can be divided into five different groups^30^,^31^. The β-lactamase enzymes encoded by bacteria have a wide substrate range, hydrolysing penicillins as well as first-, second- and third-generation cephalosporins^32^. Different groups of *bla*_CTX-M_ genes have been reported previously in *Shigella*^33^,^34^, including the two *bla*_CTX-M_ types (*bla*_CTX-M-14_ and *bla*_CTX-M-79_) found in our research. These two types belong to the CTX-M-1 group and are plasmid encoded^35^. The *S. flexneri* serotype 4s isolates that contained these two genes showed a high degree of resistance to third-generation cephalosporins compared with the other isolates (Fig. 1 and Table 3).

Quinolone levels and/or fluoroquinolone resistance correlate with mutations in the target enzymes gyrase (*gyrA* and *gyrB*) and topoisomerase IV (*parC* and *parE*), and the presence of plasmid-borne mechanisms encoded by *qnrA*, *qnrB*, *qnrS* and *aac*(*6*′)*-Ib-cr*^36^,^37^. The predominant mutations that give rise to fluoroquinolone resistance usually occur in the QRDR, from positions 67–106 of GyrA and ParC^38^. The mutation at position 83 of *gyrA* is the most frequently observed in *Shigella* species, and usually results in high-level resistance to the first-generation quinolone nalidixic acid^39^. The presence of additional mutation(s) in *gyrA* and/or another target gene such as *parC* will increase the level of resistance to fluoroquinolones^40^. In this study, all isolates had *gyrA* Ser83Leu and *parC* Ser80Ile mutations, and one strain had an additional Asp87Asn mutation in *gyrA*. Three of the strains contained *qnrS*, with two of these strains showing high resistance to levofloxacin and norfloxacin. One strain also contained *aac*(*6*′)*-Ib-cr*. Our results are consistent with the previous theory that quinolone resistance determinants alone may have a weak effect on resistance levels, but can augment resistance when combined with other determinants^41^. Moreover, the point mutation found outside of the QRDR, His211Tyr in gyrA, is very common in fluoroquinolone-resistant *Shigella*^42^. In the present research, all isolates contained the His211Tyr mutation, regardless of their fluoroquinolone sensitivities. Therefore, the way in which this mutation contributes to drug resistance should be the subject of further research.

Antibiotic resistance and the way it disseminates in *Shigella* species is related to the presence of resistance gene cassettes in class 1 and class 2 integrons^43^. In our research, we identified nine types of gene cassette distributed amongst the class 1 and class 2 integrons. The *bla*_OXA-1_ and *aadA2* cassettes in class 1 integrons are usually co-ordinately integrated, and encode resistance to ampicillin (*oxa-1*) and streptomycin (*aadA*)^44^,^45^. Strains harbouring *dfrA17* and *aadA5* cassettes were resistant to both trimethoprim and streptomycin^46^, while the *aacA4* and *cmlA1* cassettes usually confer resistance to aminoglycosides and chloramphenicol, respectively^47^. The *dfrA1*, *sat1*, and *aadA1* cassettes usually band together in class 2 integrons, and confer resistance to trimethoprim, streptothricin, and streptomycin, respectively^45^,^47^. Recent reports show that these two integrons have high prevalence rates in *Shigella* species^42^,^48^. The current findings regarding integrons in the serotype 4s isolates are consistent with these previous results, which suggest the pervasive existence of integrons, and the importance of antibiotic resistance disseminated by integrons.

It has been shown previously that the provision of clean water and good sanitation reduces the incidence of *S. flexneri* infections in people^49^. *S. flexneri* strains can survive for several months in contaminated water^50^ and foodstuffs^51^. In China, although the water and food hygiene problems are gradually improving, unsafe water and poor sanitation still exists in many areas and this is the main factor driving epidemics and outbreaks of *S. flexneri*^52^,^53^. Of concern is that most of the infections caused by *S. flexneri* 4s strains in our research resulted from the intake of contaminated water or food according to our basic epidemiological data. The persistence of *S. flexneri* in the contaminated environment also increases the chance of transmission of AMR determinants between different subtypes. We tested for the presence of multiple AMR determinants in the 4s serotype and a high prevalence rate was observed. It has been shown that most of these determinants can be obtained on multiple occasions and maintained in multiple lineages for protracted periods of time^14^. We also observed that the serotype II and Xv strains whose PFGE patterns show similarity with that of serotype 4s also exhibited high antibiotic resistance levels, while the other serotype II and Xv strains did not show such high resistance levels. Hence, we are inclined to believe that serotype 4s is horizontally transferred to serotype II and Xv strains with high antibiotic resistance, thereby transforming them into serotype 4s, rather than them being transferred along with AMR determinants into the sensitive serotype II and Xv strains. In summary, comprehensive control of the contaminated environment is important for decreasing the rate of antibiotic resistance in *S. flexneri* and for reducing its prevalence.

*S. flexneri* serotype 4s strains have shown a high level of multidrug resistance since they were first reported in 2010. In the present research, the antibiotic resistance and molecular characteristics of the 4s isolates collected from 2003 to 2013 were analysed. All of the isolates were MDR. They also had a high prevalence of antimicrobial-resistant genes and high genome diversity. The increasing drug resistance in novel *S. flexneri* serotypes has made prevention and treatment of shigellosis more difficult, and drug resistance poses a public health threat in areas where shigellosis is endemic. Because there are no reports of serotype 4s isolates outside of China, there is little concern, and therefore research, on this serotype. But the possibility of serotype 4s existing elsewhere in the world cannot be ruled out. In some parts of the world, the identification of new *S. flexneri* serotypes may be hindered by the high price of commercial antisera or the limited types of antisera available. Considering that novel serotype Xv has developed into a predominant serotype, second only to serotype 2a, in a short time in China^6^, it is important to monitor the prevalence and antibiotic resistance pattern of serotype 4s strains to enable timely intervention in the case of an epidemic. In addition, judicious use of antibiotics by physicians is needed to minimize the selection pressure of antibiotics on *Shigella* isolates. Further research should now be conducted to develop an effective vaccine against shigellosis.

## Methods

### Bacterial strains, serotyping and biochemical characterization

All of the *Shigella* strains were isolated from faecal samples from patients with diarrhoea or dysentery. The faecal samples, collected by sentinel hospitals in 13 provinces or municipalities in the eastern, western, southern, northern and central regions of China, were screened for *Shigella* species as follows. Samples were streaked onto Salmonella–Shigella (SS) agar and then incubated overnight at 37 °C. Resultant suspected *Shigella* colonies were picked and streaked directly onto SS agar and incubated overnight at 37 °C. The resultant colonies were subcultured on Luria–Bertani agar plates and grown in a 37 °C incubator. Next, the strains were submitted to our laboratory and identified to species and serotype levels. Specific serotypes were identified using monovalent antisera (Denka Seiken, Tokyo, Japan) and monoclonal antibodies (MASF IV-1 and MASF IV-2, Reagensia AB, Stockholm, Sweden). During our 13-year routine surveillance of shigellosis (from 2002 to 2014), a total of 1973 *S. flexneri* strains were isolated, and these included 24 *S. flexneri* serotype 4s isolates. API 20E test strips (bioMerieux Vitek, Marcy-L’Etoile, France) were used for biochemical characterization of the isolates following the manufacturer’s recommendations. All experiments were performed in accordance with relevant guidelines and the experimental protocols were approved by Institute of Disease Control and Prevention, Academy of Military Medical Sciences. The informed consent was obtained from all subjects.

### PFGE

The genetic relatedness of the *S. flexneri* serotype 4s isolates was analysed using *Not*I macrorestriction of genomic DNA and PFGE according to the standard protocol for *S. flexneri*, as described previously^54^. Pattern analysis was carried out using BioNumerics software version 6.0 (Applied-Maths, Sint-Martens-Latem, Belgium). The Dice coefficient of similarity was calculated with a position tolerance of 0.8%, and the dendrogram was constructed based on the unweighted-pair group method of averages.

### MLST

All isolates were assigned to multilocus sequence types using the protocols described at http://www.shigatox.net/ecmlst/cgi-bin/index. PCR amplification and sequencing of 15 housekeeping genes were performed. The primer sequences of the 15 genes utilized are available at http://www.shigatox.net/ecmlst/cgi-bin/scheme. The reactions were performed in 50-μl volumes containing 10 ng of DNA template, 0.5 mM of each primer, 1 unit of ExTaq DNA polymerase (Takara, China), 200 mM dNTPs, and 10× PCR buffer (containing 500 mM KCl, 0.1 M Tris HCl, pH 8.3), and 25 mM MgCl_2_. The PCR conditions were as follows: 94 °C for 5 min, 33 cycles of 94 °C for 30 s, 55 °C for 30 s, 72 °C for 1 min, and 72 °C for 10 min. The sequences of the 15 housekeeping genes were edited using SeqMan 7.0 and then uploaded to the EcMLST website for comparison, which allowed us to determine the ST of each gene^55^.

### Genome sequencing and phylogenetic analysis

DNA was extracted from all *S. flexneri* serotype 4s isolates using the QIAamp DNA Mini Kit (QIAGEN). Index-tagged paired-end Illumina sequencing libraries were prepared using a DNA Library Prep Kit (NEBNext® Ultra™, Illumina). The libraries were combined into pools of uniquely tagged libraries and then sequenced on the Illumina MiSeq (PE250) sequencing platform according to the manufacturer’s protocols. The core SNPs of 24 *S. flexneri* 4s strains, 35 strains from Shigella Genome Sequencing Consortium collection^14^, and 4 complete *S. flexneri* genomes (*S. flexneri* 2a 301, 2a 2457T, Xv 2002017 and 5 8401) were identified using kSNP^56^ with default parameters and a k-mer size of 13 bp. Concatenated core SNPs were then aligned for maximum likelihood phylogenetic tree construction with MEGA^57^, using the GTR model and 1000 bootstrap replicates. Phylogenetic tree of only 24 *S. flexneri* serotype 4s strains were also constructed as described above. The Whole Genome Shotgun project of *S. flexneri* serotype 4s has been deposited at GenBank under the accession LVIF00000000–LVJC00000000.

### Antimicrobial susceptibility testing

The MICs of 21 antimicrobials against the *S. flexneri* serotype 4s isolates were determined next. We used the Sensititre semi-automated antimicrobial susceptibility system (TREK Diagnostics, Inc., Westlake, OH, USA) and the Sensititre Gram-negative custom plate PRCM2F for the analysis (the antimicrobials included ceftazidime, ceftriaxone, cefepime, cefoperazone, cefazolin, cefoxitin, nitrofurantoin, imipenem, piperacillin, ampicillin, tetracycline, gentamicin, tobramycin, amikacin, aztreonam, chloramphenicol, ticarcillin, ticarcillin/clavulanic acid constant 2, levofloxacin, norfloxacin, and trimethoprim/sulfamethoxazole) according to the manufacturer’s instructions. The MICs were interpreted according to the recommendations of the Clinical and Laboratory Standards Institute. *Escherichia coli* strain ATCC 25922 was used as the quality control strain.

### Detection of antimicrobial resistance genes and integrons

Chromosomal DNA from each of the isolates was purified using a TIANamp Bacteria DNA Kit (TIANGEN Biotech, China) following the manufacturer’s recommendations. PCR assays to screen for β-lactamase genes (*bla*_VIM_, *bla*_NDM_, *bla*_SHV_, *bla*_TEM_, *bla*_OXA_, and *bla*_CTX-M_), QRDRs (*gyrA*, *gyrB*, *parC*, and *parE*), PMQR genes (*qnrA*, *qnrB*, *qnrD*, *qnrS*, and *aac*(*6*′)*-Ib-cr*), and variable regions of class 1 and class 2 integrons were performed according to previously described methods^58^,^59^,^60^. Sequences were assembled and edited using the Seqman module of the DNAstar package (DNAstar Inc., Madison, WI, USA). Nucleotide sequence similarity searches were performed using the Basic Local Alignment Search Tool from the NCBI GenBank database (http://blast.ncbi.nlm.nih.gov/Blast.cgi).

## Additional Information

**How to cite this article**: Yang, C. *et al*. Molecular characterization and analysis of high-level multidrug-resistance of *Shigella flexneri* serotype 4s strains from China. *Sci. Rep*. **6**, 29124; doi: 10.1038/srep29124 (2016).

## Supplementary Material

Supplementary Information

Supplementary Text S1

## Figures and Tables

**Table 1:** Biochemical characteristics of *S. flexneri* serotype 4s isolates.

No.	Biochemical characteristics						
	IND	GLU	MAN	SOR	MEL	ARA	others*
BJ4s01	−	+	+	−	+	+	−
BJ4s02	−	+	+	−	+	+	−
BJ4s03	−	+	+	−	+	+	−
BJ4s04	−	+	+	−	+	+	−
BJ4s05	−	+	+	−	+	+	−
GX4s01	+	+	+	−	+	+	−
GX4s02	+	+	+	−	+	+	−
GX4s03	−	+	+	−	+	+	−
GX4s04	−	+	+	−	+	+	−
GX4s05	+	+	+	−	+	+	−
HN4s01	−	+	+	−	+	+	−
HN4s02	−	+	+	−	+	+	−
HN4s03	−	+	+	−	+	+	−
HN4s04	−	+	+	−	+	+	−
HN4s05	−	+	+	−	+	+	−
HN4s06	−	+	+	−	+	+	−
SH4s01	+	+	+	+	−	+	−
SH4s02	−	+	+	−	+	+	−
SH4s04	−	+	+	−	−	+	−
SH4s05	+	+	+	−	+	+	−
SH4s06	+	+	+	−	+	+	−
SH4s07	−	+	+	−	+	+	−
SH4s08	+	+	+	−	+	+	−
SH4s09	−	+	+	−	+	+	−

IND, indole; GLU, glucose; MAN, mannitol; SOR, sorbitol; MEL, melibiose; ARA, arabinose. “others*” includes: o-nitrophenyl-β-D-galactopyranoside, arginine dihydrolase, lysine decarboxylase, ornithine decarboxylase, citrate utilization, H_2_S, urease, rhamnose, tryptophane deaminase, acetoin, gelatinase, inositol, sucrose, amygdalin.

**Table 2:** Multidrug resistance profiles of *S. flexneri* serotype 4s isolates (n = 24).

Antimicrobial resistance profiles	Antimicrobial kinds quantity	Isolates quantity	Antimicrobial classes								
			Aminog-lycosides	Cephalo-sporins	Penicillins	Quinolones	Monobactams	Amphenicols	Cephamycins	Folate pathway inhibitors	Tetracyclines
TIC/AMP/TET/CHL	4	7			+			+			+
TIC/AMP/TET/CHL/SXT	5	6			+			+		+	+
TIC/AMP/TET/CHL/CFZ/FOX	6	1			+			+	+		+
TIC/AMP/NOR/TET/CHL/SXT	6	1			+	+		+		+	+
TOB/GEN/PIP/TIC/AMP/TET/SXT	7	1	+		+					+	+
TIM/CRO/CFP/PIP/TIC/AMP/TET/CHL/CFZ	9	1		+	+			+	+		+
TIM/CRO/CFP/PIP/TIC/AMP/TET/CHL/CFZ/SXT	10	2		+	+			+	+	+	+
GEN/CRO/CFP/PIP/TIC/AMP/TET/CHL/CFZ/SXT	10	1	+	+	+			+	+	+	+
GEN/CRO/CFP/PIP/TIC/AMP/TET/CHL/CFZ/SXT/ATM	11	1	+	+	+		+	+	+	+	+
CAZ/CRO/CFP/PIP/TIC/AMP/TET/CHL/CFZ/SXT/ATM	11	1		+	+		+	+	+	+	+
CRO/CFP/PIP/TIC/AMP/LVX/NOR/TET/CHL/CFZ/SXT	11	1		+	+	+		+	+	+	+
TOB/GEN/CRO/TIC/AMP/LVX/NOR/TET/CHL/CFZ/SXT	11	1	+	+	+	+		+	+	+	+

**Table 3:** Antimicrobial resistance determinants of *S. flexneri* serotype 4s isolates.

No.	β-Lactamases gene and type			QRDR (or outside) mutation		PMQR gene		Integrons and gene cassette array	
	*bla*OXA-1	*bla*TEM-1	*bla*CTX	*gyrA*	*parC*	*qnrS*	*aac*(*6*′)*-Ib-cr*	*IntI1*	*IntI2*
BJ4s01	+	+	−	S83L, H211Y	S80I	−	−	*bla*OXA1 + aadA2	dfrA1 + sat1 + aadA1
BJ4s02	+	+	−	S83L, H211Y	S80I	−	−	*bla*OXA1 + aadA2	dfrA1 + sat1 + aadA1
BJ4s03	+	+	−	S83L, H211Y	S80I	+	−	*bla*OXA1 + aadA2	dfrA1 + sat1 + aadA1
BJ4s04	+	+	−	S83L, H211Y	S80I	−	−	*bla*OXA1 + aadA2	dfrA1 + sat1 + aadA1
BJ4s05	+	+	−	S83L, H211Y	S80I	−	−	*bla*OXA1 + aadA2	dfrA1 + sat1 + aadA1
GX4s01	+	+	*bla*CTX-M-14	S83L, H211Y	S80I	+	−	*bla*OXA1 + aadA2	dfrA1 + sat1 + aadA1
GX4s02	+	+	−	S83L, H211Y	S80I	-	−	*bla*OXA1 + aadA2	dfrA1 + sat1 + aadA1
GX4s03	+	+	−	S83L, H211Y	S80I	−	−	*bla*OXA1 + aadA2	dfrA1 + sat1 + aadA1
GX4s04	+	+	−	S83L, H211Y	S80I	−	−	*bla*OXA1 + aadA2	dfrA1 + sat1 + aadA1
GX4s05	+	+	−	S83L, H211Y	S80I	−	−	*bla*OXA1 + aadA2	dfrA1 + sat1 + aadA1
HN4s01	+	+	−	S83L, H211Y	S80I	−	−	*bla*OXA1 + aadA2	dfrA1 + sat1 + aadA1
HN4s02	+	+	*bla*CTX-M-14	S83L, H211Y	S80I	−	−	dfrA17 + aadA5	dfrA1 + sat1 + aadA1
HN4s03	+	+	*bla*CTX-M-79	S83L, H211Y	S80I	−	−	*bla*OXA1 + aadA2	dfrA1 + sat1 + aadA1
HN4s04	+	+	−	S83L, H211Y	S80I	−	−	*bla*OXA1 + aadA2	dfrA1 + sat1 + aadA1
HN4s05	+	+	−	S83L, H211Y	S80I	−	−	*bla*OXA1 + aadA2	dfrA1 + sat1 + aadA1
HN4s06	+	+	*bla*CTX-M-14	S83L, H211Y	S80I	+	+	aacA4 + cmlA1	dfrA1 + sat1 + aadA1
SH4s01	+	+	−	S83L, H211Y	S80I	−	−	*bla*OXA1 + aadA2	dfrA1 + sat1 + aadA1
SH4s02	+	+	*bla*CTX-M-14	S83L, H211Y	S80I	−	−	*bla*OXA1 + aadA2	dfrA1 + sat1 + aadA1
SH4s04	+	+	−	S83L, H211Y	S80I	−	−	*bla*OXA1 + aadA2	dfrA1 + sat1 + aadA1
SH4s05	+	+	−	S83L, H211Y	S80I	−	−	*bla*OXA1 + aadA2	dfrA1 + sat1 + aadA1
SH4s06	+	+	*bla*CTX-M-14	S83L, H211Y	S80I	−	−	*bla*OXA1 + aadA2	dfrA1 + sat1 + aadA1
SH4s07	+	+	*bla*CTX-M-14	S83L, H211Y	S80I	−	−	*bla*OXA1 + aadA2	dfrA1 + sat1 + aadA1
SH4s08	+	+	−	S83L, H211Y	S80I	−	−	*bla*OXA1 + aadA2	dfrA1 + sat1 + aadA1
SH4s09	+	+	−	S83L, S87N, H211Y	S80I	−	−	*bla*OXA1 + aadA2	dfrA1 + sat1 + aadA1
