# Supplementary material for: Molecular characterization and analysis of high-level multidrug-resistance of Shigella flexneri serotype 4s strains from China
Source: Sci Rep. 2016 Jul 4;6:29124. doi: 10.1038/srep29124 (PMC4931504; doi:10.1038/srep29124)

Molecular characterization and analysis of high-level multidrug-resistance of *Shigella flexneri* serotype 4s strains from China

Chaojie Yang, Peng Li, Xiujuan Zhang, Qiuxia Ma, Xianyan Cui, Hao Li, Hongbo Liu, Jian Wang, Jing Xie, Fuli Wu, Chunyu Sheng, Xinying Du, Lihua Qi, Wenli Su, Leili Jia, Xuebin Xu, Jiayong Zhao, Shengli Xia, Na Zhou, Hui Ma, Shaofu Qiu* and Hongbin Song*

Supplementary figure S1. SNP phylogenetic analysis of 24 *Shigella flexneri* serotype 4s strains based on the whole genomes sequences

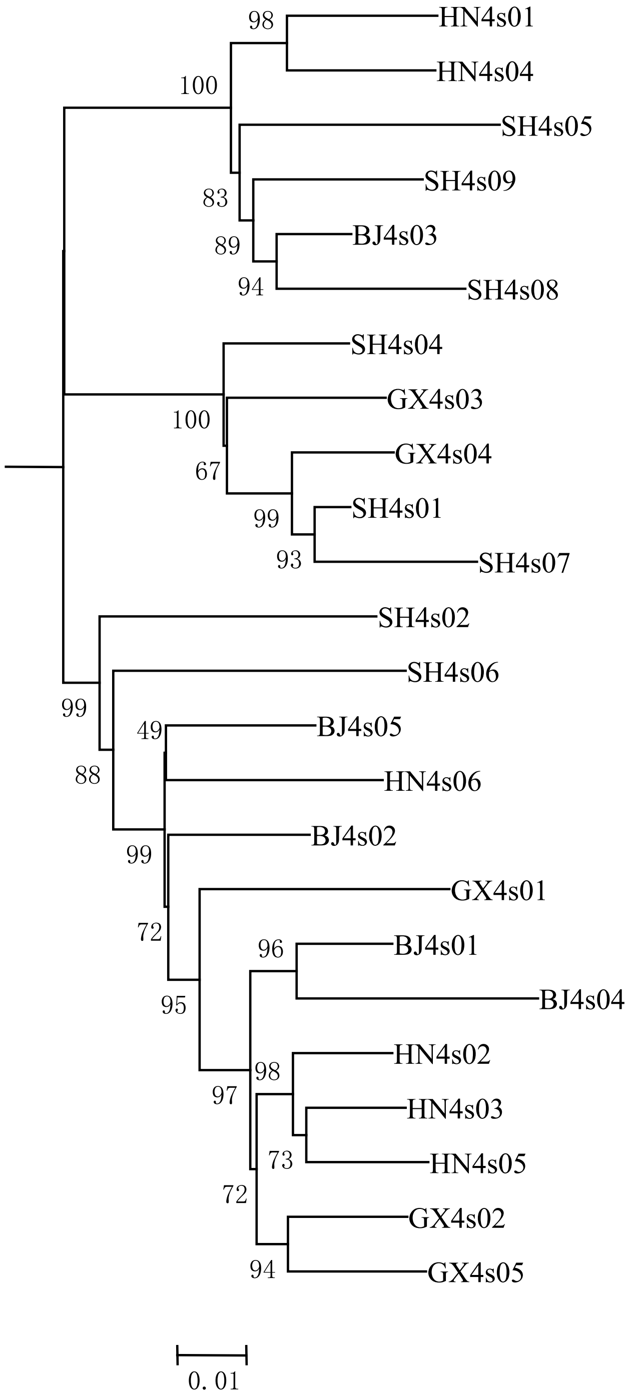

Supplement: Supplementary Information [file srep29124-s1.pdf]
