# Supplementary material for: Molecular characterization and analysis of high-level multidrug-resistance of Shigella flexneri serotype 4s strains from China
Source: Sci Rep. 2016 Jul 4;6:29124. doi: 10.1038/srep29124 (PMC4931504; doi:10.1038/srep29124)
Supplement: Supplementary Text S1 [file srep29124-s2.pdf]

Molecular characterization and analysis of high-level multidrug-resistance of *Shigella flexneri* serotype 4s strains from China

Chaojie Yang, Peng Li, Xiujuan Zhang, Qiuxia Ma, Xianyan Cui, Hao Li, Hongbo Liu, Jian Wang, Jing Xie, Fuli Wu, Chunyu Sheng, Xinying Du, Lihua Qi, Wenli Su, Leili Jia, Xuebin Xu, Jiayong Zhao, Shengli Xia, Na Zhou, Hui Ma, Shaofu Qiu* and Hongbin Song*

Supplementary Text S1.
New DNA sequences and new allele combination of MLST result in this research.

New DNA Sequences:
Allele Name: rpoS
Isolate GX4s03:
ATTGCCCGCCGTTATGGCAATCGTGGTCTGGCGTTGCTGGGCCTTATCGAAGAGGGCAACTT
GGGGCTGATCCGCGCGGTAGAGAAGTTTGACCCGGAACGTGGTTTCCGCTTCTCAACATACG
CAACCTGGTGGATTCGCCAGACGATTGAACGGGCGATTATGAACCAAACCCGTACTATTCGT
TTGCCGATTCACATCGTAAAGGAGCTGAACGTTTACCTGCGAACCGCACGTGAGTTGTCCCA
TAAGCTGGACCATGAACCAAGTGCGGAAGAGATCGCAGAGCAACTGGATAAGCCAGTTGATG
ACGTCAGCCGTATGCTTCGTCTTAACGAGCGCATTACCTCGGTAGACACCCCGCTGGGTGGT
GATTCCGAAAAAGCGTTGCTGGACATCCTGGCCGATGAAAAAGAGAACGGTCCGGAAGATAC
CACGCAAGATGACGATATGAAGCAGAGCATCGTCAAATGGCTGTTCGAGCTGAACGCCAAAC
AGCGTGAAGTACTGGCACGTCGATTCGGTT
Isolate GX4s01:
ATTGCCCGCCGTTATGGCAATCGTGGTCTGGCGTTGCTGGACCTTATCGAAGAGGGCAACTT
GGGGCTGATCCGCGCGGTAGAGAAGTTTGACCCGGAACGTGGTTTCCGCTTCTCAACATACG
CAACCTGGTGGATTCGCCAGACGATTGAACGGGCGATTATGAACCAAACCCGTACTATTCGT
TTGCCGATTCACATCGTAAAGGAGCTGAACGTTTACCTGCGAACCGCACGTGAGTTGTCCCA
TAACACGTGAGTTGTCCCATAAGCTGGACCATGAACCAAGTGCGGAAGAGATCGCAGAGCAA
CTGGATAAGCCAGTTGATGACGTCAGCCGTATGCTTCGTCTTAACGAGCGCATTACCTCGGT
AGACACCCCGCTGGGTGGTGATTCCGAAAAAGCGTTGCTGGACATCCTGGCCGATGAAAAAG
AGAACGGTCCGGAAGATACCACGCAAGATGACGATATGAAGCAGAGCATCGTCAAATGGCTG
TTCGAGCTGAACGCCAAACAGCGTGAAGTACTGGCACGTCGATTCGGTT

New allele combination:
Isolate SH4s05:

Allele Name	arcA	aroE	aspC	clpX	cyaA	dnaG	fadD	grpE	icdA	lysP	mdh	mtlD	mutS	rpoS	uidA
Number	8	10	13	16	10	11	14	10	16	23	19	15	14	64	14
